# Supplementary material for: Respiratory symptoms and cardiovascular causes of deaths: A population-based study with 45 years of follow-up
Source: PLoS One. 2022 Oct 20;17(10):e0276560. doi: 10.1371/journal.pone.0276560 (PMC9584444; doi:10.1371/journal.pone.0276560)
Supplement: S1 Table — (PDF) [file pone.0276560.s001.pdf]

S1 Table. Descriptive statistics for participants at different stages according to response to questionnaires.

	All (nonresponse, response), with start date		Response to smoking status		Previous column + response to education		Previous column + response to occupational exposure		Previous column + response to respiratory symptoms		Previous column + response to 3 items on heart disease	
	No.	%	No.	%	No.	%	No.	%	No.	%	No.	%
Age, years												
15–29	18212	12	12783	11	12391	11	12083	11	11832	11	11115	12
30–44	59158	38	40417	35	39511	35	38561	36	37817	36	35924	38
45–59	42985	27	32617	29	31985	29	30640	29	29613	29	27161	28
≥60	36541	23	28563	25	28237	25	26191	24	24619	24	21504	22
Sex												
Male	122734	78	89143	78	87614	78	84361	78	81510	78	75483	79
Female	34162	22	25237	22	24510	22	23114	22	22371	22	20221	21
Highest attained education												
Compulsory education (<11 years)				26	29513	26	27354	25	25950	25	23358	24
Medium level (11-13 years)				52	58200	52	56140	52	54390	52	50239	52
University level (>13 years)				22	24411	22	23981	22	23541	23	22107	23
Smoking status												
Never			38699	34	37774	34	36103	34	34916	34	32583	34
Previous			30676	27	30214	27	28993	27	27986	27	25313	26
Current			45005	39	44136	39	42379	39	40979	39	37808	40
No. of cigarettes per day												
0–9			16521	24	16229	24	15502	24	14957	24	13662	24
10–19			34238	50	33710	50	32510	50	31583	50	29020	50
≥20			18140	26	17755	26	17230	26	16682	26	15261	26
Occupational exposure gas/dust												
Yes			48376	44	47509	44	47509	44	45563	44	41503	43
No			61232	56	59966	56	59966	56	58318	56	54201	57
Cohort study												
Oslo 72	19892	13	17680	15	17377	15	16445	15	16084	15	15421	16
Hordaland 85	4982	3	4404	4	4347	4	4307	4	4137	4	3785	4
Støvlunge 88–90	108812	69	76673	67	75406	67	71958	67	69168	67	63540	66
Oslo/Hordaland 98–99	23210	15	15623	14	14994	13	14765	14	14492	14	12958	14
Birth cohort												
1895–1919	17686	11	13816	12	13697	12	12515	12	11733	11	10462	11
1920–1929	23735	15	19279	17	19075	17	17927	17	17036	16	15160	16
1930–1939	24546	16	19168	17	18827	17	18000	17	17322	17	15691	16
1940–1949	37160	24	26932	24	26257	23	25486	24	24869	24	23363	24
1950–1959	37475	24	25190	22	24654	22	24068	22	23637	23	22530	24

1960–	16294	10	9995	9	9614	9	9479	9	9284	9	8498	9
Angina pectoris												
No			101939	96	99937	96	96217	96	93658	96	93096	97
Yes			4123	4	4061	4	3823	4	3490	4	2608	3
Other heart disease												
No			102711	98	100693	98	96940	98	94274	98	93972	98
Yes			2452	2	2406	2	2278	2	2139	2	1732	2
Myocardial infarction												
No			102445	97	100412	97	96642	97	94028	97	93340	98
Yes			3653	3	3609	3	3428	3	3153	3	2364	2
N	156896		114380		112124		107475		103881		95704	
