# Supplementary material for: Respiratory symptoms and cardiovascular causes of deaths: A population-based study with 45 years of follow-up
Source: PLoS One. 2022 Oct 20;17(10):e0276560. doi: 10.1371/journal.pone.0276560 (PMC9584444; doi:10.1371/journal.pone.0276560)
Supplement: S2 Table — (PDF) [file pone.0276560.s002.pdf]

S2 Table. Prevalence of baseline respiratory symptoms overall and in never smokers according to cardiovascular causes of death.

	Total sample		According to cardiovascular cause of death									
			Acute myocardial infarction		Other ischemic heart disease		Other heart disease		Cerebrovascular		Other circulatory	
	No.	%	No.	%	No.	%	No.	%	No.	%	No.	%
Overall												
No. of breathlessness symptoms												
0	78132	82	2879	70	1529	66	1695	76	1922	75	820	73
1	9584	10	554	13	345	15	255	11	309	12	136	12
2	5634	6	439	11	284	12	214	10	201	8	107	10
3	1752	2	200	5	136	6	64	3	89	4	40	4
4	602	1	51	1	32	1	16	1	32	1	17	2
No. of cough/phlegm symptoms												
0	63014	66	2636	64	1431	62	1509	67	1732	68	720	64
1	17030	18	717	17	418	18	359	16	402	16	176	16
2	6891	7	356	9	219	9	149	7	165	7	87	8
3	4052	4	182	4	112	5	101	5	127	5	60	5
4	2709	3	119	3	82	4	71	3	74	3	49	4
5	2008	2	113	3	64	3	55	3	53	2	28	3
No. of asthma/wheeze symptoms												
0	73130	76	2996	73	1649	71	1723	77	2007	79	808	72
1	16465	17	849	21	477	21	397	18	403	16	234	21
2	6109	6	278	7	200	9	124	6	143	6	78	7
Never-smokers												
No. of breathlessness symptoms												
0	28684	88	800	77	399	70	591	80	674	83	205	83
1	2168	7	97	9	72	13	69	9	69	8	21	8
2	1273	4	92	9	67	12	58	8	51	6	15	6
3	343	1	44	4	28	5	14	2	19	2	6	2
4	115	0.4	4	0.4	6	1	4	1	2	0.2	1	0.4
No. of cough/phlegm symptoms												
0	24684	76	816	79	428	75	573	78	646	79	197	79
1	5035	15	142	14	74	13	97	13	103	13	32	13
2	1402	4	35	3	43	8	33	4	29	4	10	4
3	769	2	25	2	16	3	18	2	21	3	6	2
4	419	1	10	1	8	1	6	1	11	1	2	1
5	274	1	9	1	3	1	9	1	5	1	1	0.4
No. of asthma/wheeze symptoms												
0	28301	87	886	85	476	83	627	85	722	89	213	86
1	3088	9	117	11	73	13	81	11	74	9	27	11
2	1194	4	34	3	23	4	28	4	19	2	8	3
