# Supplementary material for: Respiratory symptoms and cardiovascular causes of deaths: A population-based study with 45 years of follow-up
Source: PLoS One. 2022 Oct 20;17(10):e0276560. doi: 10.1371/journal.pone.0276560 (PMC9584444; doi:10.1371/journal.pone.0276560)
Supplement: S3 Table — Hazard ratios with 95% confidence intervals and p-values according to cause of death, multivariable proportional hazards regression analysis adjusted for sex, education, occupational exposure to gas/dust and birth cohort (n = 90,316). (PDF) [file pone.0276560.s003.pdf]

S3 Table. After exclusion of patients with known heart disease. Hazard ratios (HR) with 95% confidence intervals and p-values according to cause of death, multivariable proportional hazards regression analysis adjusted for sex, education, occupational exposure to gas/dust and birth cohort (n=90316).

	All CV		Acute MI		Other ischemic heart		Other heart		Cerebrovascular		Other circulatory	
	HR	95%CI	HR	95%CI	HR	95%CI	HR	95%CI	HR	95%CI	HR	95%CI
Breathless on effort, score (vs. 0)												
1	1.31***	[1.23,1.40]	1.29***	[1.15,1.45]	1.50***	[1.30,1.75]	1.17*	[1.00,1.37]	1.37***	[1.19,1.57]	1.21	[0.98,1.50]
2	1.59***	[1.47,1.72]	1.52***	[1.32,1.75]	1.89***	[1.58,2.27]	1.70***	[1.42,2.03]	1.37***	[1.14,1.64]	1.60***	[1.24,2.06]
3	1.98***	[1.74,2.26]	2.05***	[1.64,2.57]	2.73***	[2.06,3.62]	1.53*	[1.08,2.17]	1.83***	[1.36,2.45]	1.74*	[1.12,2.70]
4	1.76***	[1.40,2.22]	2.14***	[1.48,3.08]	1.78*	[1.03,3.08]	1	[0.49,2.04]	1.76*	[1.05,2.94]	1.9	[0.95,3.79]
Cough and phlegm, score (vs. 0)												
1	0.97	[0.92,1.03]	1.01	[0.92,1.11]	1	[0.87,1.14]	1	[0.88,1.14]	0.93	[0.82,1.04]	0.87	[0.73,1.05]
2	1.03	[0.95,1.11]	1.06	[0.93,1.21]	1.18	[0.99,1.41]	1.04	[0.86,1.25]	0.88	[0.74,1.06]	0.88	[0.68,1.13]
3	1.09	[0.99,1.20]	0.98	[0.82,1.17]	1.01	[0.80,1.28]	1.16	[0.92,1.47]	1.29*	[1.05,1.57]	1.1	[0.82,1.49]
4	1.01	[0.90,1.14]	0.83	[0.67,1.04]	1.05	[0.80,1.38]	1.26	[0.95,1.66]	1.04	[0.80,1.36]	1.17	[0.82,1.65]
5	1.07	[0.93,1.23]	1.08	[0.85,1.36]	0.99	[0.72,1.37]	1.37	[0.99,1.88]	0.98	[0.71,1.36]	0.82	[0.51,1.31]
Attacks of breathlessness and wheeze, score (vs. 0)												
1	0.97	[0.92,1.03]	1.02	[0.92,1.12]	1	[0.88,1.15]	0.98	[0.86,1.12]	0.85*	[0.74,0.96]	1.05	[0.88,1.26]
2	0.93	[0.85,1.03]	0.92	[0.78,1.09]	1.09	[0.89,1.35]	0.86	[0.68,1.08]	0.81	[0.65,1.01]	1.11	[0.83,1.49]

* p<0.05, ** p<0.01, *** p<0.001
