# Supplementary material for: Respiratory symptoms and cardiovascular causes of deaths: A population-based study with 45 years of follow-up
Source: PLoS One. 2022 Oct 20;17(10):e0276560. doi: 10.1371/journal.pone.0276560 (PMC9584444; doi:10.1371/journal.pone.0276560)
Supplement: S4 Table — Hazard ratios with 95% confidence intervals and p-values according to cause of death, multivariable proportional hazards regression analysis adjusted for sex, education, occupational exposure to gas/dust and birth cohort (n = 72,339). (PDF) [file pone.0276560.s004.pdf]

S4 Table. After exclusion of patients with known heart disease or lung disease. Hazard ratios with 95% confidence intervals and p-values according to cause of death, multivariable proportional hazards regression analysis adjusted for sex, education, occupational exposure to gas/dust and birth cohort (n=72339).

	All CV		Acute MI		Other ischemic heart		Other heart		Cerebrovascular		Other circulatory	
	HR	95%CI	HR	95%CI	HR	95%CI	HR	95%CI	HR	95%CI	HR	95%CI
Breathless on effort, score (vs. 0)												
1	1.33***	[1.24,1.44]	1.30***	[1.14,1.49]	1.44***	[1.20,1.72]	1.27**	[1.06,1.52]	1.40***	[1.19,1.64]	1.22	[0.95,1.56]
2	1.65***	[1.50,1.82]	1.51***	[1.26,1.80]	1.92***	[1.54,2.40]	1.69***	[1.35,2.12]	1.49***	[1.20,1.85]	1.93***	[1.45,2.58]
3	2.14***	[1.81,2.54]	2.40***	[1.80,3.20]	2.42***	[1.64,3.57]	1.37	[0.84,2.24]	2.07***	[1.42,3.02]	2.38***	[1.42,3.99]
4	2.04***	[1.40,2.97]	1.38	[0.61,3.11]	2.70*	[1.26,5.82]	1.04	[0.33,3.30]	3.75***	[2.01,6.99]	0.79	[0.11,5.74]
Cough and phlegm, score (vs. 0)												
1	0.97	[0.91,1.04]	0.98	[0.88,1.10]	0.94	[0.81,1.10]	0.99	[0.85,1.15]	0.98	[0.85,1.12]	0.91	[0.73,1.05]
2	1.01	[0.93,1.11]	0.99	[0.84,1.16]	1.23*	[1.01,1.51]	0.95	[0.76,1.20]	0.9	[0.73,1.11]	0.96	[0.68,1.13]
3	1.11	[0.99,1.25]	1.02	[0.82,1.26]	0.89	[0.65,1.21]	1.11	[0.83,1.50]	1.42**	[1.12,1.80]	1.16	[0.82,1.49]
4	1	[0.86,1.17]	0.81	[0.60,1.08]	1.17	[0.84,1.63]	1.24	[0.87,1.77]	0.85	[0.58,1.24]	1.32	[0.82,1.65]
5	0.98	[0.80,1.20]	0.81	[0.55,1.20]	0.95	[0.59,1.51]	1.46	[0.95,2.25]	1.05	[0.68,1.63]	0.65	[0.51,1.31]
Attacks of breathlessness and wheeze, score (vs. 0)												
1	0.97	[0.90,1.03]	1.01	[0.90,1.14]	1.09	[0.93,1.27]	0.94	[0.80,1.11]	0.84*	[0.72,0.98]	0.97	[0.78,1.20]
2	0.96	[0.83,1.10]	0.85	[0.65,1.10]	1.22	[0.91,1.64]	1.01	[0.72,1.41]	0.87	[0.63,1.20]	0.97	[0.63,1.49]

* p<0.05, ** p<0.01, *** p<0.001
