# Supplementary material for: Respiratory symptoms and cardiovascular causes of deaths: A population-based study with 45 years of follow-up
Source: PLoS One. 2022 Oct 20;17(10):e0276560. doi: 10.1371/journal.pone.0276560 (PMC9584444; doi:10.1371/journal.pone.0276560)
Supplement: Attachment — Submitted filename: 26sep2022_response_to_reviewers_pone_v2.docx [file pone.0276560.s005.docx]

PONE-D-22-14710

Response to reviewers

*Thanks for the many comments from the academic editor and the three reviewers that we think have contributed to improving our paper. Please find point-to-point responses to the comments.*

**Academic editor**

I think the aim is relevant – to determine whether respiratory symptoms increase the mortality (risk) of different cardiovascular diseases. Keep the manuscript to that aim!!

*We have kept that focus.*

Delete all the primary analyses regarding SES, occupational exposures, education, etc., etc. They are also of interest, of course, but in another paper. But of course, keep them as potential confounders, but do not present primary mortality estimates

*We have removed Table 3 (old manuscript), with the crude death rates, as suggested, including the corresponding lines 220-223 in the text.*

The table are too many, and difficult to interpret. If you skip the primary analysis of the confounders (and just mention that the analyses are adjusted for occupation, etc, etc), then you can merge some of the tables.

*In Tables 4,5,6,7,8 (old manuscript), we have kept the lines with respiratory symptoms, and removed the lines with the confounders. The revised table headings state what variables we have adjusted for.*

*We have also removed the annotations in the text to the effect of these confounders, i.e.., old manuscript lines 229-236 and 253-255, 257-259, 268-269, 274-276, 295, 296-299 and 308-309.*

*The stratified analyses of men and women have been combined to one table (new Table 4).*

*The analysis of total sample exclusive those with heart disease have been moved to the supplement (new manuscript, S3 Table, in supplement).*

*Finally, we have added another table to the supplement (as “data not shown” line 320 in old manuscript is not allowed with PlosOne); new S4 Table in the supplement.*

*The questionnaire (S1 Table old manuscript has been inserted as new regular Table 2 in the new manuscript, as suggested by reviewer #2).*

*Missing references have been inserted.*

*Finally, we have removed death due to “lung embolism” as an endpoint in the analyses, see comment to reviewer #2.*

**Editorial office**

**Supplementary comments to the editorial office**

(1) **Request for additional details regarding ethical consent**

This has been elaborated on, with reference to a previous publication (24) and inserted in a new section, Ethical approval and consent, lines 106-132:

*Regional committees for Medical Research Ethics were established in Norway in 1985 [26]. Therefore, for the first survey cohort in 1972 (Oslo 72) there was no requirement for ethical approval. Answering and returning the questionnaire was considered as an informed consent.*

*The participants of the Hordaland County cohort 1985 survey (Hordaland 85) and the Hordaland and Oslo counties cohort 1998-1999 survey (Oslo/Hordaland 98–99) all signed an informed consent, which was approved by the Regional Ethical Committee of Health Region West [24].*

*The attendants of the Hordaland County and Sauda municipality in Rogaland County 1988-1990 survey (Støvlunge 88–90) were invited to X-ray surveillance for tuberculosis where attendance is demanded by Norwegian law. The consent information described the health examination, including risk of technical procedures and blood collections, as well as data protection and genetic assessment. Participants could ask questions about the examination, and they were required to give written consent before any part of health examination was conducted [24].*

*The Norwegian Data Inspectorate allowed the use of unique personal identification numbers to gather all information collected in the four surveys on each individual and to link information from Statistics of Norway on death, causes of deaths and highest attained education. The Norwegian Directorate of Health and Social Services granted us permission to receive information on notification of causes of deaths. Finally, the Data Inspectorate, the Norwegian Directorate of Health and Social Services and Regional Ethical Committee granted permission to have a common data registry for all surveys, for all follow-up studies, and the specific causes of deaths [24].*

24. Gulsvik A, Humerfelt S, Bakke PS, Omenaas ER, Lehmann S. Norwegian population surveys on respiratory health in adults: objectives, design, methods, quality controls and response rates. Clin Respir J. 2008;2 Suppl 1:10-25. Epub 2008/10/01. doi: 10.1111/j.1752-699X.2008.00080.x. PubMed PMID: 20298346.

26.	Lahlum E, Ruyter KW. The establishment of ethical committees for medical research. Tidsskr Nor Laegeforen. 2012;132(12-13):1486-9. Epub 2012/07/07. doi: 10.4045/tidsskr.12.0311. PubMed PMID: 22766826.

(2) **Revised data availability statement**

A data custodian has been added, in addition to the first author. The Ethics committee does not provide data access and has been omitted.

**Reviewer #1**

Please consider the suggestions listed below each section:

Abstract:
Line 3: Consider to replace the word causes with diseases in this sentence to be more clear.

*Done*

Line 6 : Add “by using”: cardiovascular deaths by using Cox regression analysis……

*Done*

Line 25 Please consider rephrasing the abstract conclusion to be even more precise. If I am not mistaken the study did not assess if breathlessness is an early marker of CV deaths, but whether breathlessness is associated with CV deaths (as stated in the aim). Alternatively, consider stating state: Breathlessness could be an early marker of CV deaths? (or similar).

*We have rephrased the conclusion, as suggested*

Overall: Please omit the space between number and %: 18% instead of 18 %.
*This is a matter of style, and we do not think there is a consensus on this in English; however, we have omitted the space throughout the manuscript and tried to be consistent. (In contrast, a space is recommended in Norwegian)*

Introduction:
Line 45: Please provide a reference for this statement.

*We have added two missing references*

Line 57: should this be CV deaths?

*That is correct; CV “has” been added!*

Line 62: consider writing: that were not adjusted for…

*Revised sentence, as suggested: “, and the analyses were not adjusted for”*

Methods:
The details of the methodology are considered to be sufficient to allow the experiments to be reproduced. Nevertheless authors are encouraged to include some brief information in the manuscript regarding the follow-up time, although a reference (19) is provided. Information is included in the follow-up and census data section, but what was the range or the shortest follow-up time?

*We had previously presented median and maximum follow-up. The shortest was 1 day (0.00274 years), which has been added. Also, errors in some numbers have been corrected in the last sentence of the paragraph.*

Results:
The results and tables are well presented and easy to understand for the reader.
Line 229: please omit the bracket after word sex.

*Removed sentence, see comment to academic editor.*

Line 234: please consider commenting this finding in the discussion. Could it be due to a healthy worker effect?

*We agree that this is possible. However, this information has been deleted in the revision, ref. comments from the academic editor leading to smaller tables without showing the confounders. This is not applicable (N/A) after revising the tables.*

Line 245: should this sentence read: myocardial infraction declined with increasing score of breathlessness and wheeze?

*Agree; this has been corrected*

Line 258: Please state the direction of this association (higher education seems to be protective).

*N/A after revising the tables*

Line 287: Please insert “was” and “stratified analyses”: lung embolism was excluded in the stratified analyses due to few persons.

*Thanks, this has been corrected*

Line 296: same as above.
analyses due to few persons.

*Thanks, this has been corrected*

Line 300 to 302: Please consider rephrasing this sentence as it is a bit difficult to understand.
*Agree. This has been revised*

Line 308: Please consider defining early death.
*N/A after revising the tables*

Discussion:
The discussion is balanced and well written, please find some minor suggestions below
Line 361: insert the word “were”: The respiratory
questions were more or less identical to those in the present study.
*Done*

Line 373: should this read: previous cardiopulmonary disease?

*Yes; corrected.*

Line 384: sample size of that study?

*N/A (Lung embolism has been removed from the manuscript)*

Line 398: Please consider replacing “are” with “is”: a causal verification is lacking.

*Done (as well as corrected “or” to “of”)*

Line 403: Replace “have” with “has”.

*Done*

Line 417: is a reference missing here”?

*Agree. Two missing reference have been inserted here..*

Line 438-439 The meaning of this sentence: “However, the variability of response…” is not quite clear, please adjust.

*Lines 438-440 have been rephrased:*

*“However, the prevalence of respiratory symptoms and the reproducibility of the responses tend to vary by smoking habits and educational level, and the prevalence rates may be influenced by small changes in the phrasing of the questionnaire items (27)”.*

An original data deposited in not made available in repositories due to ethical limitations placed by the Regional Committees for Medical and Health Research Ethics in Norway. A minimal data set can be made available for scientific analysis on request, provided that the respective research institution proofs handling of the data strictly in accordance with ethical regulations (written ethics protocol, full compliance with the Declaration of Helsinki).
The study seems to conform well with the STROBE guidelines.

**Reviewer #2**

I have some questions about the article:
The authors should consider some language review since the grammar is questionable throughout the manuscript.
Other main areas of concern are considering the tables, which are too large, not self-explanatory and include too much information with limited value (the “other” headings and more). More information is needed concerning the scales used.

*The tables and the corresponding text have been adjusted, see comments to the academic editor above.*

*The tables have been reduced, and there are fewer in the main paper.*

Abstract:
p2 row 6: insert the word “using”. …using cox analysis

*Done, see also comment to reviewer #1*

2. Information is needed in the abstract about what scale has been used for assessing breathlessness that has been used (maybe on row 5). (an adaption of the mMRC). As it is now, it is unclear what score three and score two mean. Rows 14-24 are a little vague.
Introduction

*We have dropped “Score 1” and “Score 3” from the abstract and have specified that the questionnaire was a modification of the MRC questionnaire on chronic bronchitis. In the revised paper the questionnaire is presented as a new Table 2.*

1. P4 row 46
2. Should write chronic obstructive pulmonary disease instead of lung disease?

*Done*

3. Row 50 “No studies are available of studies of” … , strange language.

*The sentence has been revised: “There is little information available...”*

Methods results and discussion:

1) The section about the study population is hard to follow and understand. Could it be clearer to use some flow diagram or graphic?

*We have previously tried to prepare flow diagrams, but concluded that a table was more feasible here (Table 1)*

2) I need more information concerning the scale used to assess breathlessness. The question asked is vital to interpreting the results. My suggestion is that the table presented in the supplementary material should be included in the manuscript.

*Done (new Table 2).*

3) It is hard to find more thorough information on the “Norwegian respiratory questionnaire” online or elsewhere; the cited sources do not really give any more information. Especially citation 24. However, some information can be found in citation 22. However, this manuscript would benefit from a more comprehensive description of the questionnaires since the symptom questions are the backbone of this study.

*See comment above. The questionnaire was shown as a Supplementary Table, but has now been included as a regular Table. The questionnaire has previously been presented in ref. 20,21,22,24,26 in the new manuscript (these references have been inserted).*

4) I lack information on how many were lost to follow-up? ( it is said that 231 emigrated without any available date, but how many were lost all in all? ).

*All Norwegians have a nation person ID (PIN) and all death certificates are linked with this register. The register would have complete information from available death certificates for people listed as living in Norway. In the present study, to our knowledge 231 persons were lost to follow up because of emigration and were censored in the analyses. For persons listed as living in Norway, the register states that they cover about 98% of deaths in Norway (see also the next point, where more information has been inserted in the manuscript). The persons in our analyses are clearly given in Table 1.*

5) More information is needed in the manuscript concerning establishing the cause of death. A smaller section is found in the discussion, but the information is also required in the methods section. (see also points 6-9)

*This has been expanded and more details provided, new manuscript lines 176-183:*

*“The Norwegian cause of death registry has directly identifiable data on individuals from 1951 [Gjertsen]. It covers about 98% of all deaths in Norway, and most missing values are for people dying abroad. Until 2005 the underlying cause of death was determined manually by the coding personnel at the Cause of Death Registry. From 2005 the underlying cause of death has been determined electronically by a software application (Automated Classification of Medical Entities, ACME) developed by the National Center for Health Statistics in the US). The listed causes of death on the death certificate are first translated manually into ICD-10 codes, and then the ACME software determines the underlying cause of death (31-33)”*

6) What is the validity of the registry of causes of death in this setting? How are they obtained? (through autopsy or clinically only?) Has any previous study examined the agreement between the registered cause of death and the “real” cause? (information needed in the methods section and not only in the discussion)

*Added to methods (see also above), new manuscript lines 200-207:*

*“Death certificates are the only source of information on death in >90% of cases. The autopsy rate in Norway had declined over time. Autopsy rates in Norway have fluctuated over time.*

*In the early 1960 it was about 10% of all deaths, increasing to 18% in 1986 and gradually declining to slightly above 7% in 2010 (Vollset SE, Tell GS, 2012, in: Vollset et al, 2012:). The rate was between 7 and 9% from 2011 to until 2019 (https://gateway.euro.who.int/en/hfa-explorer/#USmrpBXlVB). The diagnostic validity of cerebral stroke and ischemic heart disease as underlying cause of death in this registry showed a substantial agreement between mortality statistics and autopsy findings, although there is a selection for post-mortem examinations [Gulsvik AK et al].”*

7) Starting on row 338, the authors discuss pulmonary embolism as a cause of death; it seems like the authors had expected more cases. But what would an expected number of deaths attributed to pulmonary embolism be? How common should death from PE be in an average population? The authors argue that better diagnostics would potentially lead to a higher number, but at the same time argue that the causes of death registry are reliable. Suppose the causes of death are not established by autopsy. In that case, it is plausible that most actual pulmonary embolisms would be categorised as something else (perhaps as a myocardial infarction) by the clinician responsible for the death certificate. This finding then raises concerns about the actual reliability of the causes of the death registry in Norway and then also raises concerns about the outcome variables in this study.

8) Should pulmonary embolism be removed and only mentioned briefly in the text? I do not find that it gives that much important information as it is now – mainly since cases are very few and there are questions concerning its validity.

*We agree that lung embolism is a difficult cause-of-death in a registry, both in hospitalized and non-hospitalized subjects. Despite having a large sample of several pooled cohorts, the number of deaths due to lung embolism in our study was small. This is also reflected in the literature, as few studies would have statistical power to investigate determinants of deaths attributed to lunge-embolism. However, we think that the validity of lung embolism as an underlying cause of death might be lower than for some others diagnoses, and after some consideration, we have simply decided to remove lung embolism as a cause of death in the paper.*

*In the discussion, we have added a sentence on this, revision, lines 424-426.:*

*“We initially also analyzed on pulmonary embolism as a cause of death. However, because of few events, changes in diagnostic procedures over time and presumed challenges with validity of the diagnosis, we decided to leave this out of the paper.”*

9) It is unclear if only one cause of death was used in this material? Were secondary or tertiary causes of death available? How was the leading cause of death established? If several simultaneous reasons were present, how was this handled?

*Only the underlying cause of death was used, as stated in Methods. The underlying cause of death is always only one cause, see e.g. definition from CDC webpage:*

*The underlying cause-of-death is defined by the World Health Organization (WHO) as "the disease or injury which initiated the train of events leading directly to death, or the circumstances of the accident or violence which produced the fatal injury." Underlying cause-of-death is selected from the conditions entered by the physician on the cause of death section of the death certificate. When more than one cause or condition is entered by the physician, the underlying cause is determined by the sequence of conditions on the certificate, provisions of the ICD, and associated selection rules and modifications*

[*https://wonder.cdc.gov/wonder/help/ucd.html*](https://wonder.cdc.gov/wonder/help/ucd.html)

*See also comments on the procedures at the registry (above)*

In my view, myocardial infarction is often stated as the primary cause of death even when there is no known cause; the actual underlying condition is often more interesting but is often expressed as a secondary or tertiary cause of death. This would jeopardise the reliability of the outcomes. For example, if an individual with severe pulmonary neoplasm in palliative care ultimately died from a cardiovascular condition, wouldn’t the pulmonary neoplasm be the most relevant cause of death? Any thoughts about how this was handled?

*This should be clearer from the revised paragraphs inserted in the Materials and Methods chapter, providing more details. The Norwegian register follows similar procedures as other registries for ascertaining the underlying cause of death.*

*We think it is beyond the scope of the paper to dive more into this. For more information, please see:*

<https://tidsskriftet.no/2000/03/kronikk/dodsarsaksregisteret-og-forskning>

<https://www.fhi.no/hn/helseregistre-og-registre/dodsarsaksregisteret/dodsarsaken-kodes-med-icd-koder/>

<https://www.fhi.no/globalassets/dokumenterfiler/helseregistre/dar/dodelighet-og-dodsarsaker-pdf.pdf>

10) On the same note, even though this study focuses on cardiovascular disease, it would be interesting to see the proportions of different causes of death in the population. Was cardiovascular disease prevalent in an expected ratio of all deaths?

*We think that giving the proportion of deaths in the persons of our survey population is beyond the scope of this paper (see also comments from the academic editor about removing the crude death rates). It is also complicated to compare the death rates in our cohorts with the total population with proper adjustments for age, birth cohort and year of death, as detailed information is not readily available. Mortality from CV disease in Norway changed considerably over time during the last 50 years, with a peak around 1970 and a rapid decrease until 2010.*

*However, the rates of CV deaths in the cohort in the present study of overall 353 (per 100.00 person-years) is in crude agreement with National Norwegian data of CV deaths from 1951 to 2010 (1970: about 950 for men, 650 for women, 2010: 300 for men, 200 for women). (Folkehelseinstituttet rapport ISBN 978-82-8082-524-7, 2012 4, p. 15), reference #34 in the paper.*

11) It is unclear to me how adjustments were handled and selected. Were all factors really adjusted for the same variables? As this is a very complex analysis, it is plausible that there is a complex interplay between different factors that could include confounding effects on the analyses. Performing a structured analysis of possible confounders could be of great importance. (such as http://www.dagitty.net/ ). There is a considerable risk that confounders could influence these results, which should be addressed thoroughly. More information on the adjustments is needed within the tables, preferably so that one can see precisely which factors are adjusted for what.

*See the revised tables with revised titles/footnotes and the comments to the academic editor. The procedure for the models is also described in methods, underscoring that we adjusted for the same variables across models. We do not think creating a post-hoc directed acyclic graph is a good idea.*

12) Could results be visualised better? Kaplan Mayer or cumulative incidence curves?

*The focus here is on the hazard rates. Plots of the Cox models or Kaplan-Meier curves will contain overlying curves and CIs are hard to present properly on top of this. We would also be careful with cumulative incidence curves and want to abstain from complicating the analysis with competing risk analyses here. We think tables are the best way of presenting these data.*

13) More work is needed with the tables; they are too large, a lot of information is lacking, and I believe they include too much information. (see my points 13-16 for details)

*We have reduced the tables in the revised version, see comments to academic editor.*

14) More information is needed within the tables (such as explanations of abbreviations, headings, detailed information on adjustments etc.). The tables are also too large. The tables should stand on their own. As it is now, the reader must always refer to the text to understand the different headings.

*See the previous point.*

15) I am not sure that the headings “other heart”, “other vascular”, and “other ischemic” are needed within the tables. The valuable information from such categories is limited, and I suggest that these are removed with the added benefit of more readable tables. Then there is no need to specify the different headings within the table, which I believe is otherwise needed. The “other” headings are not even referred to in the text. Some explanation on why these are included and what information the authors believe they can get from them is needed in case they are not removed.

*We do not quite understand this point.* *The labels used refers to those used in Table 3, with the codes used for grouping/classifying underlying causes of death.*

16) What are “myocardial infarction – yes/no” and “other heart disease yes/no” as stated in the tables? Is it a previous myocardial infarction/heart disease? What is the value of having these added to the tables?

*These are questionnaire items that the participants reported at inclusion, which was shown in details in Methods including phrasing of the items (Questionnaire, last paragraph). If they had a history of previous heart disease, we think this would be important information. This could be associated with both respiratory symptoms and the CV outcome of interest.*

17) Could educational level be dichotomised in some way to merge the categories?

*In the revision, we have focused on the association of the respiratory symptoms with CV death, and used this for adjustment in the models. We a priori chose to use three educational levels and have therefore*

*kept three levels in the models, although the HRs for the confounders are now not shown. See comments to academic editor.*

18) The row “Total” in table 3 is strange. What is meant by this? Is there not a total score for each of the objects within the table – then why choose to include only one? My suggestion is to remove it.

*This seems to be a misunderstanding. This is not scores at all, but crude numbers of deaths. The Total row was the crude number of deaths for each outcome and 100% (the sum of deaths for men and women). However, we have removed Table 3 altogether, see comments to academic editor.*

19) Row 389-399: Interesting discussion, but it seems these rows would be better in another section of the manuscript, perhaps below “Main findings” instead, as these are discussed.

*We have moved row 389-401 (old manuscript), as suggested.*

**Reviewer #3** 
Thank you for the opportunity to review the manuscript: ” Respiratory symptoms and cardiovascular causes of deaths: a population-based study with 45 years of follow-up” by Knut Stavem et al.

The manuscript is well written and discusses an important question on breathlessness symptoms and risk for CVD - death in a large population. These symptoms are very frequent in clinic and these results emphasize the importance of investigation of the causes of breathlessness.
There are some concerns as follows:

In methods: There is a large number of individuals that weren't included in the final analyses. A drop from 158702 individuals to final 95704 was registered. Possible impact of this drop-out should be discussed as a weakness in this study.

*We agree and have added a comment to the discussion to address this (new manuscript, lines 442-446):*

*“The 95,705 individuals included in the final analyses represented about 60% of the target population, as we included only individuals with responses to a number of questionnaire items on possible confounding variables. This may be a weakness of the study, as there is a risk that non-participations bias. However, it is hard to delineate how this will impact associations between respiratory symptoms and CV deaths.”,*

Moreover, the lack of information on diseases increasing risk for cvd-death as diabetes, and hypertension is also a weakness with this study not mentioned by authors.

*We have expanded a paragraph in the discussion to address this, lines 429-433:*

*“It is well known that CV deaths are associated with sex, birth cohort, smoking, occupational exposure to particles, educational level and diagnosis of CV and pulmonary diseases [53, 54]. The present study has adjusted for these variables. However, we did not adjust for other known CV risk factors, such as diabetes, obesity, lipids, hypertension, diet, psychological factors, or lung function, which were not available in our study.”*

The start of the observation: It is not clear to me why the authors didn’t choose the date of filling or left questionnaires for start date of the observation

*Added (new manuscript, lines 101-104):*

*“The start of the observation was the date of returning the fill out self-administered questionnaire to the survey center. However, the actual date was not available in two of the cohorts. In one cohort we had the month and year of participation, and in the fourth we used a fixed date approximately when invitations were sent.”*

*(Details for the imputation of missing start dates are described under statistical analysis)*

It is not quite clear what were the procedures in the inclusion for each cohort and whether the questionnaires were filled home or in situ in the study center? Did they have any visit or help on filling in the questionnaires? This should be stated in the methods.

*Added (new manuscript, lines 87-89):*

*“The random sampling was conducted by the national Population register using a random number generator.”*

*Added (new manuscript, lines 95-96)*

*“In all surveys, we used a self-administered questionnaire filled out at home by the person invited without any help of persons from the survey center.”*

Results: In general, the results are somehow difficult to follow because of the amount of information and tables. The authors should consider concentrating on results important on answering the research question and in specific breathlessness and cvd-mortality. Some of the tables can be part of supplementary material including table 7 and 8.

*Agree. The tables have been reduced and some unnecessary information left out. Please see comments to the academic editor as well.*

In Table 7, it is difficult to understand how many events there are in each strata. I suggest a column with information on event for each row.

*The overall numbers of deaths according to CV causes are shown in new S2 Table (in supplement); however, not for the sub-strata. Adding these numbers to all the stratified tables would add another 5 columns to each table and lead to very large tables. Instead, we have added the number of events for never-smokers (which was requested) to the bottom of this new S2 Table.*

Discussion: End of the first paragraph” The associations increased with the severity of breathlessness, and the pattern of associations was similar in women and in never smokers”- This is not true as in most of the analyses it wasn’t shown that having 4 symptoms was associated with higher risk. Consider re-writing that.

*The paragraph has been revised to address this, lines 345-347.*

*“The associations increased with the severity of breathlessness until score 3, and the pattern of associations was similar in women and in never smokers”*

*In addition, we have added the following (new manuscript, lines 367-370):*

*“That there was less association of the CV outcomes between breathlessness score 4 than score 3, might be related to one of the questions “Are you breathless when at rest?”. It is possible that this item captures other types of deconditioning than those related to CV risk, such as anxiety and hyperventilation”*
